# Severe secondary hyperkalemia and arrhythmia from drug interactions between calcium‐channel blocker and voriconazole: a case presentation

**DOI:** 10.1186/s12882-021-02370-6

**Published:** 2021-05-10

**Authors:** Xinju Zhao, Chunyan Zhang, Li Zhu, Bei Wu, Yun Han, Michael Heung, Li Zuo

**Affiliations:** 1 Department of Nephrology, Peking University People’s Hospital, Beijing, China; 2 Department of pharmacy, Peking University People’s Hospital, Beijing, China; 3 Division of Nephrology, Department of Medicine, University of Michigan, Ann Arbor, MI USA

**Keywords:** Acute kidney injury, Hyperkalemia, Arrhythmia, Drug interaction, Calcium‐channel blockers

## Abstract

**Background:**

Patients with kidney disease may have concurrent hypertension and infection. Dihydropyridine calcium-channel blockers (CCB) are the most popular class of antihypertensive drugs used in clinical settings and can be metabolized by cytochrome P450 isoenzyme 3A4 (CYP3A4). Voriconazole is a commonly used antifungal treatment and a CYP3A4-inhibitor. Insufficient attention to drug interactions from the concomitant use of CCB and voriconazole may result in serious adverse reactions.

**Case presentation:**

Here, we report a patient with acute kidney injury on stable anti-neutrophil cytoplasm antibody associated vasculitis who developed hyperkalemia resulting in sinus arrest with junctional escape rhythm attributed to drug interactions of CCB with voriconazole. This is a very rarely reported case and may be an under-recognized complication. After continuous renal replacement therapy and changing the anti-hypertensive drugs, symptoms, and laboratory abnormalities of the patient fully recovered.

**Conclusions:**

This case warns us of severe consequences of drug interactions. Co-prescription of CYP3A4-inhibitors with calcium-channel blockers increases the risk of hypotension and acute kidney injury, which may further induce hyperkalemia and arrhythmia.

## Background

Concurrent hypertension and infection are common for patients with either acute or chronic kidney disease (CKD), especially for those who are under immunosuppressant therapy. The prevalence of hypertension ranges from 60 to 90 % depending on the stage of CKD and its cause [1, 2]. Dihydropyridine calcium-channel blockers (CCB) have added greatly to the therapeutic options for treatment of hypertension and can be metabolized by cytochrome P450 isoenzyme 3A4 (CYP3A4) [3]. Voriconazole, which is a CYP3A4-inhibitor, is the first-line therapy for several severe fungal diseases, such as invasive aspergillosis [4, 5]. CCB and voriconazole may therefore be co-administrated occasionally. CYP3A4-inhibitors can raise the concentration of CCB and cause hypotension and even acute kidney injury (AKI) [6–10].

Here, we report a rare case of serious adverse reactions from excessive potentiation of calcium-channel blocker via synergistic CYP3A4 inhibition by voriconazole. The patient developed hypotension, acute kidney injury (AKI), hyperkalemia, and sinus arrest with a junctional escape rhythm. Though she gained full recovery, we would like to share the experience and warn all practitioners of these important drug interactions.

## Case presentation

**Fig. 1:**
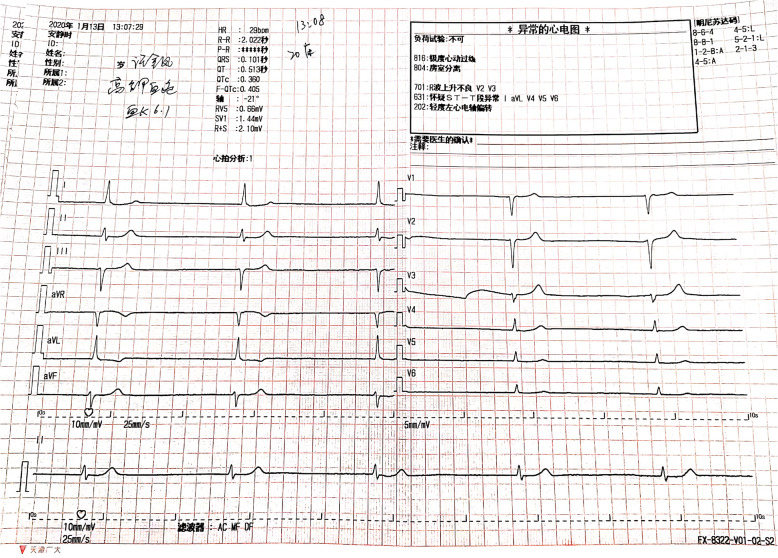
Twelve-Lead electrocardiogram revealed sinus arrest with junctional escape rhythm

**Fig. 2:**
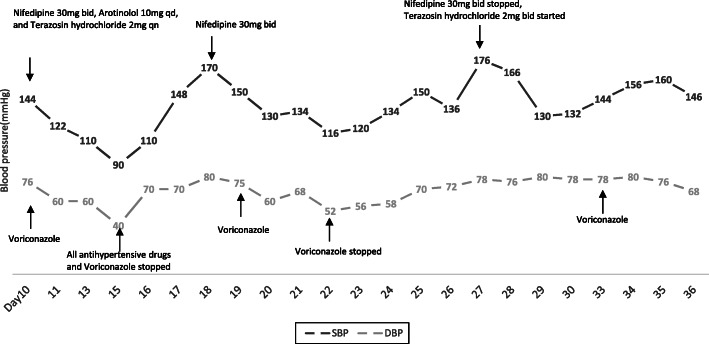
The changes of blood pressure and clinical data

A 69-year-old woman with newly diagnosed anti-neutrophil cytoplasm antibody (ANCA) associated vasculitis (AAV) was hospitalized for cough and mild shortness of breath. A renal biopsy 4 weeks before she was admitted to the hospital showed fibrocellular crescent nephritis and coexistence of membranous nephropathy. She was treated with a combination of glucocorticoids and cyclophosphamide to induce remission of her new-onset organ-threatening AAV. In detail, she was prescribed intravenous methyl-prednisone 500 mg daily for 3 days and then reduced to 50 mg daily; cyclophosphamide (0.4 g) was administrated intravenously every 2 weeks. The patient’s serum creatinine (Scr) level decreased from a peak of 428.9umol/L (4.85 mg/dl) to 305 umol/L at time of admission. She also had hypertension and had been prescribed nifedipine 30 mg BID, arotinolol 10 mg daily, and terazosin hydrochloride 2 mg nightly since AAV onset a month ago. Her blood pressure was about 140/80mmHg under antihypertensive therapy. Her heart rate (HR) was around 60 bpm with sinus rhythm. The urine flow cytometer analysis revealed that red blood cells were 106/ul, and white blood cells were 18/ul. Her pulmonary computed tomography (CT) scan showed interstitial lesions with multiple exudations. Aspergillus flavus was cultivated from her sputum. Hence, oral voriconazole was administrated beginning on day 10 on admission. On day 12 of admission, the patient felt fatigued and her blood pressure (BP) was 120/70mmHg. Myocardial-specific isoenzyme of creatine kinase (CK MB) and cardiac troponin I(CTNI), hemoglobin, serum creatinine and electrolytes were reevaluated on that day. CK MB and CTNI were normal. Her Scr was 272 umol/L (3.08 mg/dl), potassium 4.43umol/L, and hemoglobin dropped from 95 g/l to 78 g/l with no sign of blood loss. The counts for white blood cells and platelets were stable. Erythropoietin was prescribed. On day 13 of admission, the patient continued to feel fatigued and a little breathless; her blood pressure was 110/60mmHg. Her urine output was reduced to 600ml and 500ml on day 13 and 14. On day 15 of admission, the patient had a total weight gain of 6 kg from admission and her serum potassium was found to be 6.08umol/L, Scr was 340 umol/L, and the calculated blood nitrogen: Scr ratio was 22. The electrocardiography (ECG) revealed sinus arrest with a junctional escape rhythm and a HR of 29 bpm (Fig. 1). Her BP dropped to 90/40mmHg (Fig. 2) and all anti-hypertensive drugs were stopped.

## Differential diagnosis

The first step in developing a differential diagnosis was to determine the cause of hypotension. The first consideration was due to anti-hypertensive therapy together with salt restriction in the hospital. However, the treatment regimen had been used for a month, for which the BP changes were unreasonable and pushed us to keep looking for unrevealed reasons. Other causes of hypotension were under consideration, such as septic shock, cardiogenic shock, and hypovolaemic shock. However, the patient had no fever. Her respiratory symptoms were mild, and she needed no supplemental oxygen. Her echocardiogram revealed normal ventricular wall motion with preserved ejection fraction. Her history of present illness and physical examination showed no signs of volume depletion. On the contrary, she gained weight. So, all the three causes of hypotension were ruled out.

Adrenocorticotropic hormone (ACTH) and serum cortisol levels and rhythms were also tested to exclude adrenal insufficiency. The results revealed abnormal serum cortisol rhythms (Table 1). The patient was taking oral prednisone every morning which might influenced the 8am serum cortisol. Through these tests, we excluded severe adrenal insufficiency. Later, we learned that voriconazole may induce Cushing syndrome and subsequent adrenal insufficiency when administered concurrently with corticosteroids [11, 12]. This patient’s acute clinical process and the results of serum cortisol levels did not support this situation.

**Table 1:** Adrenocorticotropic hormone and cortisol levels

	0 am CST	8 am CST	4pm CST
ACTH (pg /ml)	3.8 (no reference range)	7.7 (7.2–63.3)	4.9 (no reference range)
Serum cortisol (ug/dl)	10.96 (no reference range)	3.67 (6.7–22.6)	3.31 (0–10)

We considered that hypotension was the main cause of this AKI episode. Other causes of AKI were also carefully ruled out, such as whether the kidney injury was due to other prerenal conditions, intrinsic kidney injury, or obstructive uropathy. The ultrasound for her renal arteries and veins was normal. The CT scan for the urinary system was normal. Her ANCA titer was lower than before and she was under intensive treatment for remission induction and the Scr was down-trending before this episode. Therefore, this episode of AKI could not be attributed to the new formation of crescents in the kidneys. There was no risk factor identified for interstitial nephritis. The platelet counts remained on the similar level, and no elevated lactate dehydrogenase and total bilirubin were observed, and the free hemoglobin was within normal range which suggested that there was no clinical evidence for thrombotic microangiopathy.

The renal tubular injury could not be ruled out totally. Increased risk of AKI has been observed in patients undergoing treatment with voriconazole [5]. Therefore, voriconazole was also stopped.

## Treatment of severe hyperkalemia and AKI

**Fig. 3:**
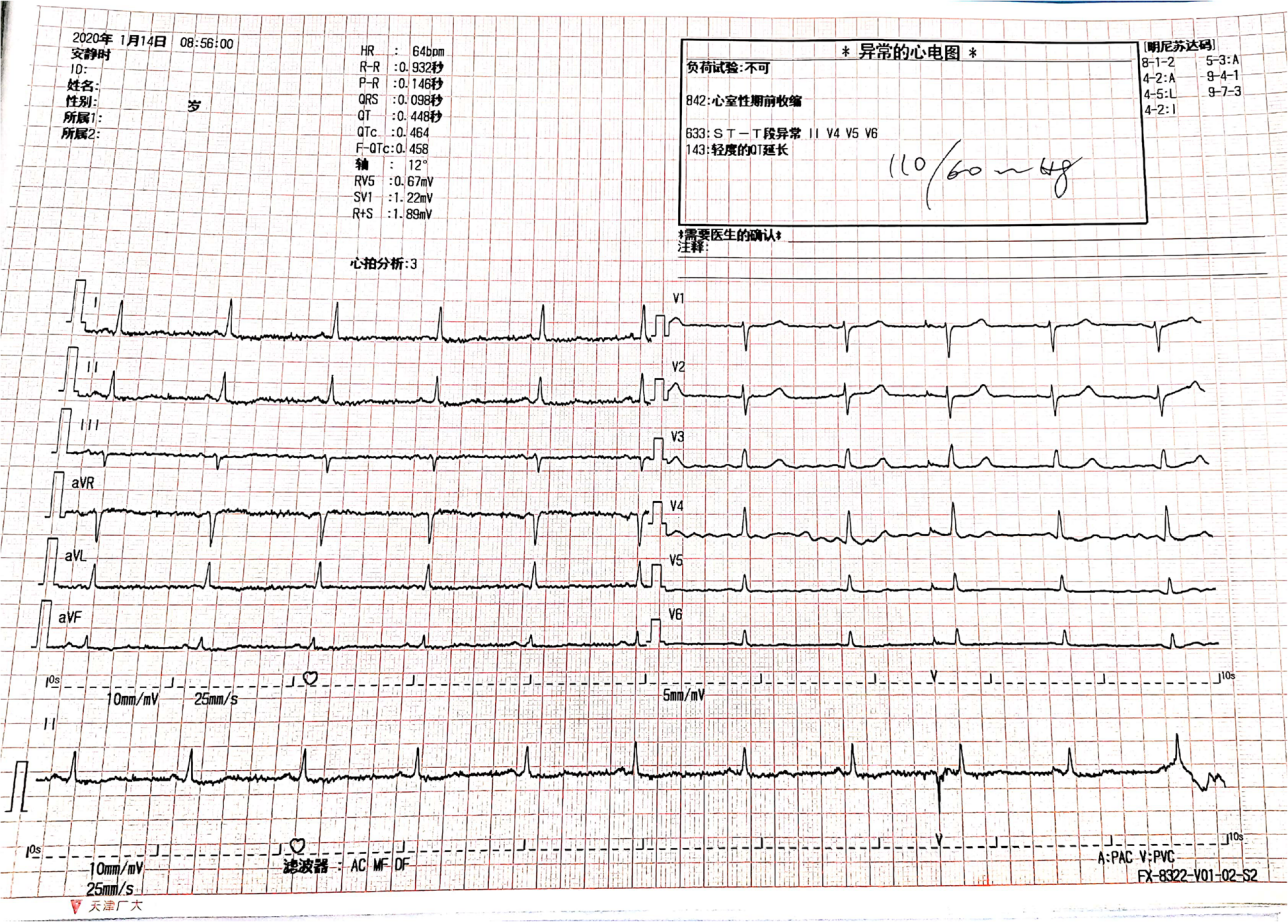
Twelve-lead electrocardiogram during sinus rhythm

**Fig. 4:**
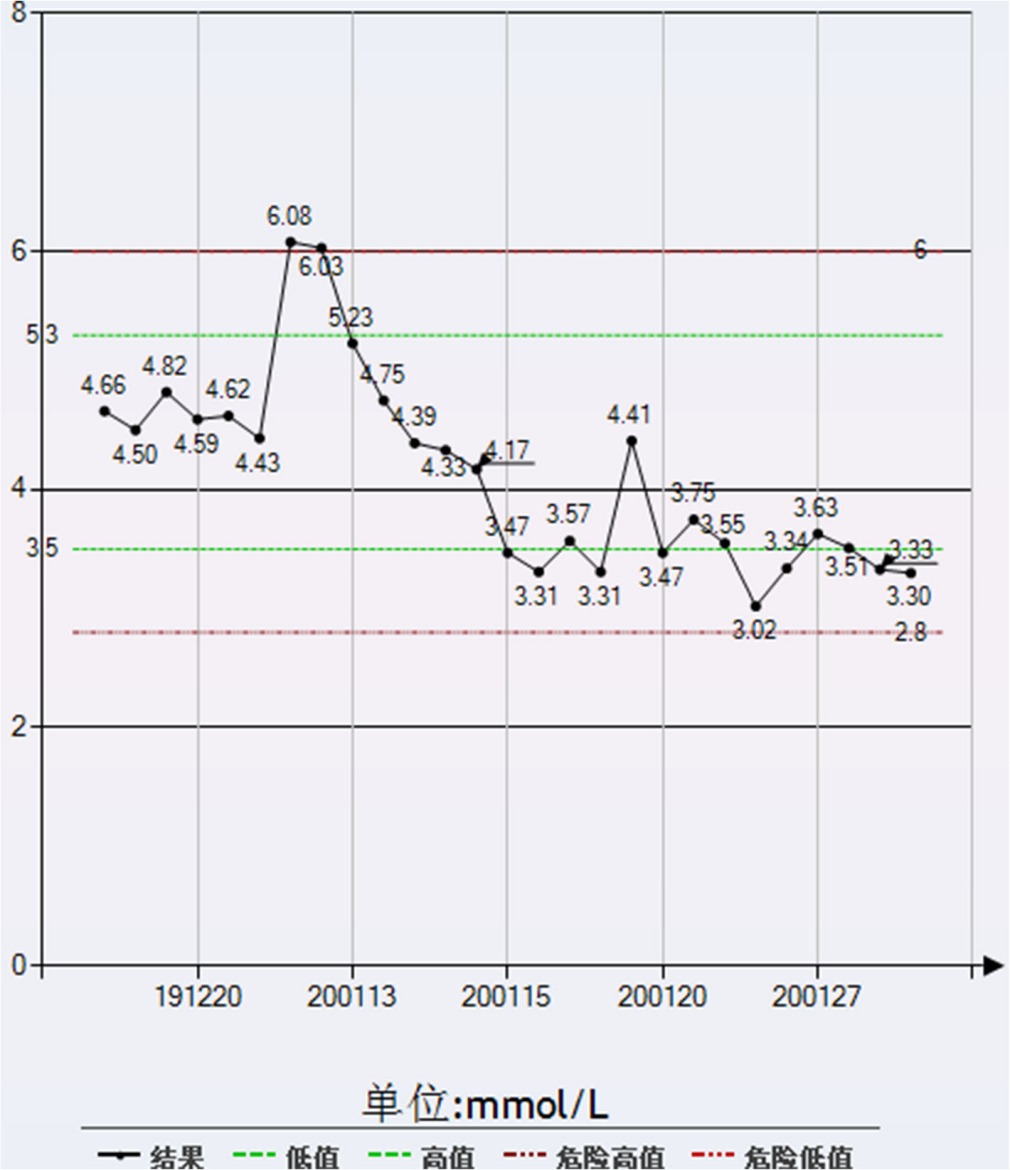
The changing course of serum potassium levels

Norepinephrine was administrated immediately. We also prescribed intravenous calcium gluconate, insulin-glucose infusion, and oral calcium polystyrene sulfonate at once. The electrophysiologist from our hospital suggested temporary pacemaker placement. However, the patient was relatively circulatory stable. More importantly, her arrythmia should be secondary to hyperkalemia which we could reverse within a couple of hours. Concerned with these, a non-cuffed catheter was placed in her femoral vein at the bedside. Continuous renal replacement therapy (CRRT) was provided for 22 h starting on day 15 of admission. On day 16 of admission, she recovered to normal sinus rhythm (Fig. 3) with a heart rate of 60 bpm, with a BP of 110/70 mmHg, and her potassium gradually went down to 4.17 umol/L (Fig. 4). Her urine output improved from 100ml on day 14 t0 800 ml on day 18, and her BP rebounded as high as 170/90 mmHg. So, nifedipine 30 mg bid was resumed. On day 19 on admission, the urine output was 900 ml and voriconazole was restarted. The patients’ urine output subsequently decreased dramatically and the blood pressure fell to between 115–134/52-68mmHg.

For this second episode of anuria onset, we realized that decreased urine output certainly had some connection with voriconazole usage. So, voriconazole was stopped and the patients’ urine output gradually increased to normal and hemodialysis was discontinued. Her Scr varied from 210 to 290 umol/L.

Through reviewing the case, reading references, and communicating with pharmacists, we hypothesized that the episode of hypotension was induced by the drug interaction of CCB and voriconazole. We determined that she should use antihypertensive drugs that do not metabolize from CYP3A4. So, when her blood pressure rose again to 170/90mmHg, she was prescribed terazosin hydrochloride 2 mg BID. Voriconazole was resumed without incident. She was under medical observation for 3 more days and her condition remained stable prior to discharge from the hospital.

## Follow‐up

Two months after her discharge from the hospital, her Scr was 220 umol/L with normal serum potassium and urine output. Her blood pressure was 140/76mmHg. Her AAV has stayed in remission and she has not had any further episodes of hyperkalemia or arrhythmia.

## Discussion and conclusion

We present a case of a life-threatening adverse reaction from excessive potentiation of CCB via CYP3A4 inhibition by voriconazole. The patient developed hypotension and AKI, and this was followed by hyperkalemia and sinus arrest with a junctional escape rhythm.

Voriconazole, a commonly used antifungal triazole, has potent inhibitory effects on CYP3A4 [5]. In the presence of CYP3A4-inhibitors, drugs that are metabolized by CYP3A4, such as nifedipine, will accumulate leading to toxicity [6, 10]. Hypotension from co-prescribing voriconazole together with nifedipine has been reported [6, 10]. Hypotension induced by concomitant use of CCB and macrolide antibiotics, particularly erythromycin and clarithromycin, are more frequently reported [7–9]. Like in our case, voriconazole can raise the blood concentration of nifedipine and excessively potentiate its hypotensive effect, resulting in ischemic AKI through renal hypoperfusion [10]. The nifedipine concentration should be high in our patient’s blood. Unfortunately, the nifedipine concentration could not be tested in our institute. Furthermore, there are significant individual variances in the disposition of nifedipine, and genetic factors are considered to play an important role [13, 14].

Nifedipine is a vessel dilator and in overdose the effective blood volume could be lower than normal. Hence, the kidney will retain sodium and water to compensate for the decreased effective blood volume. In our case, the patient retained 6Kg fluids to maintain the circulating blood volume. These consequences are consistent with a previously reported case, in which 4Kg fluids were retained [10].

Before the arrhythmia occurred, the patient’s blood pressure was 110–120/70, which seemed not too low. This also happened in the previous case, and the authors speculated that it was because of impaired kidney autoregulation on top of impaired kidney function [10]. The impaired kidney is much more vulnerable to relative hypotension than the normal kidney which may further predispose to AKI.

In our case, hyperkalemia followed AKI. Then sinus arrest with junctional escape rhythm and low HR followed hyperkalemia which in turn undermined the cardiac output and blood pressure, hence further decreasing kidney perfusion. This vicious cycle was broken by CRRT which corrected the patient’s hyperkalemia and allowed the cardiac rhythm to recover to normal sinus rhythm. CRRT also presumably reduced the accumulated nifedipine which helped to ameliorate patient’s hypotension.

Our case occurred using oral voriconazole. Some studies report intravenous voriconazole is associated with more episodes of AKI, because of the cyclodextrin vehicle, sulphobutylether-beta-cyclodextrin, which is cleared by the kidney and has been associated with nephrotoxicity [15].

This rare case warns us of the severe consequences of drug interactions. One should always be very careful when co-administrating medications, and we suggest clinicians consult pharmacists if possible. As shown in our case, co-prescription of CYP3A4-inhibitor with CCB increases the risks for hypotension and AKI, and may cause severe safety issues.

## Data Availability

The dataset supporting the conclusions of this article is included within the article. Other regarding data and material can be obtained from the first author.
